# Design of planar distributed three beam electron gun with narrow beam separation for W band staggered double vane TWT

**DOI:** 10.1038/s41598-020-80276-3

**Published:** 2021-01-13

**Authors:** Cunjun Ruan, Pengpeng Wang, Huafeng Zhang, Yiyang Su, Jun Dai, Yikun Ding, Zheng Zhang

**Affiliations:** School of Electronics and Information Engineering, Beihang University, Beijing, 100191 China

**Keywords:** Engineering, Optics and photonics, Physics

## Abstract

A novel planar distributed three-beam electron gun with narrow beam separations is designed based on grids loaded sheet beam method. The dimensions of the three-beam gun in the y–O-z plane are determined using our basic theoretical design method developed for sheet beam gun. The results show that the profile of focusing electrode in the y–O-z plane is related to the beam width in the x-O-z plane. Then, the characteristics and parameters of three-beam array formation with their stability are analyzed thoroughly by adjustment of control grids in the x-O-z plane. Each of the beamlet obtained is with a small axial deviation of the two transverse waists. Based on the theoretical analysis and simulations, the planar three-beam electron gun is constructed with the beam voltage of 22 kV and the current of 3 $\times$ 0.15 A. The average radius of 0.08 mm at each beam waist is obtained with the compression factor of 4 for the 0.18 mm beam tunnel radius. The beam waist can be achieved at about 4.4 mm away from the cathode with the axis separation about 0.46 mm for each of beamlet. Thus, the design method can be generally used to construct such type of narrow beam separation and planar distributed multiple beam electron gun for the miniaturization and integrated vacuum electron devices in millimeter wave and terahertz band.

## Introduction

High-power millimeter-wave and terahertz (THz) sources play a critical role in a wide spectrum of potential applications, including high-data-rate communications, biomedical diagnosis, chemical spectroscopy and threat detection^1–3^. Travelling wave tube (TWT) is a kind of vacuum electron devices (VEDs) which can provide high output power in a broad bandwidth^4^. However, the millimeter-wave or THz TWT combined with conventional circular beam is difficult to fulfill these demands due to the limitation of beam current carrying capacity. The adoption of a sheet beam can significantly promote the beam’s current capacity, but the successive problems are difficulty in sheet beam focusing and an over-mode beam tunnel^5,6^, which may also result in beam self-excitation and oscillation. However, planar distributed multi-beam TWT, with independent of beam tunnels and convergent beamlets, can achieve high output power by sustaining the total high beam current with a small current for each beam, which may avoid the over-mode beam tunnels comparing with the sheet beam devices. Moreover, the planar interaction circuit suitable for the planar distributed multi-beam array is not only easier in fabrication than the circular structures in the millimeter wave and THz regime, but also it can meet the trend of planarity, miniaturization and integration for next generation of vacuum electron devices^7,8^.

Focused on the numerous advantages of integrated VEDs with planar multi-beam array, there is an increasing number of groups engaged in related research. The planar multi-beam arrays are not only used well in the folded waveguide TWT^9–12^, but also used in the staggered double vane TWT^13,14^. Up to now, there are three kinds of planar multi-beam devices with unique interaction characteristics between the beamlets and the high-frequency field. Firstly, each beamlet matches an independent waveguide, which is identical to the single tube and operates in the fundamental mode. Thus, the adjacent axial separation in this kind of device is not limited and can be adjusted according to the level of fabrication. There are two typical works as following. In 2010, a 220 GHz three-beam cascaded serpentine TWT is developed^10^. The simulation using MAGIC-3D shows that the cascaded three-beam device can achieve a high-gain of 42 dB with a very compact circuit length of only 1.5 cm. As part of the DARPA HiFIVE program, Northrop Grumman Corporation developed a 220 GHz five-beam folded waveguide TWT in 2013 based on power combination among the five identical FWG circuit with finite gain^9^. The short pulse tests resulted in 56 W at 214 GHz and 5.0 GHz bandwidth. However, the spacing distance between the adjacent beam axes in both of the two devices is large enough to construct an independent electron gun for each beamlet. Thus, it is easy to obtain the well-converged beam array by a similar approach in multi-beam klystron^15^. Secondly, two or three beamlets may match a common interaction circuit using over-moded structure^11,14^, e. g, the adjacent axial separation is about 1 ~ 2 mm for the over-moded multi-beam devices for W-band high power coherent radiation. Thirdly, two or three beamlets match a common interaction circuit, which operates in the fundamental mode^12,13^. The adjacent axial separation is, generally, less than 0.5 mm for 95 GHz and 0.2 mm for 220 GHz high output power tubes. Thus, for the latter two cases, it is challenging to develop new multi-beam gun design techniques to generate the desired planar multi-beam array with so narrow beam separation.

The present work in this paper is a part of our project study of “*W*-band Multiple Beam High-power Staggered Double-vane TWT”^13^. Since the designed staggered double-vane slow wave structure (SWS) operates in the fundamental mode (${\text{TM}}_{11}$) under the first spatial harmonic, the spacing between adjacent beam axes is so narrow that is only 0.46 mm. To decrease the emission current density and keep the narrow beam separation, we’d like to construct a novel planar three-beam gun that combines the sheet beam gun with the special control grids^6^.

In this paper, a novel planar distributed three-beam electron gun with narrow beam separations is theoretically designed based on grids loaded sheet beam method, which have been develop by our group recently^15^. This design method is a universal method which has been fully verified in reliability and stability to obtain planar electron beam by one-dimensional compression of cathode electron beam. Moreover, we have also analyzed and verified the stability of the designed electron gun, including the sensitive dependence on the geometric size, and some electric parameters such as voltage and current. Therefore, this novel design method can be widely used in millimeter wave and terahertz planar electron beam devices. Thus, compared with the relevant literature^15,16^, a synthesized new method, which combines the theoretical analysis and numerical calculation is firstly adopted to design the novel planar three-beam gun with a narrow beam separation. Due to the plane symmetry of the three-beam array, the gun is designed the same as the profile of sheet beam gun with a whole throughout, which is different comparing with the conventional design concept of paraxial approximation for the structure construction with multiple beamlets^16,17^. Then, the dimensions and the position of the control grids are adjusted combined with the construction of a special focusing electrode to generate the desired planar three-beam array with the narrow beam separation. The good agreements have been achieved between the theoretical analysis and 3D simulation for the designed planar three-beam electron gun.

## Design specifications and methodologies

**Figure 1:**
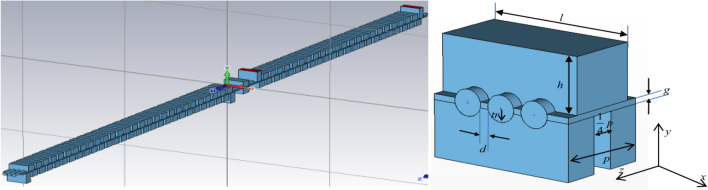
Schematic of W-band three-beam staggered double-vane TWT with the single period of the structure.

The design W-band three beam high-power staggered double-vane (SDV) TWT with the scheme of a single period structure is given in Fig. 1, which shows the arrangement of the planar three electron beam with its SDV SWS. Thus, the design specifications of the planar three-beam electron gun are as follows. The operating voltage $U_{a}$ and the beam current *I* are 22 kV and 3 $\times$ 0.15 A, respectively. Each of the beam tunnel radii is 0.18 mm and the axis separation of the adjacent electron beams is 0.46 mm. The three-beam array is characteristic with so narrow beam separation due to the space limitation in the x-axis of the fundamental mode, which will result in a high emission current density and a shrunk cathode lifetime based on conventional one circular beam electron gun. Thus, a novel planar distributed three-beam gun based on grids loaded sheet beam gun is developed, and the guidelines of design methodologies are presented in the paper.

**Figure 2:**
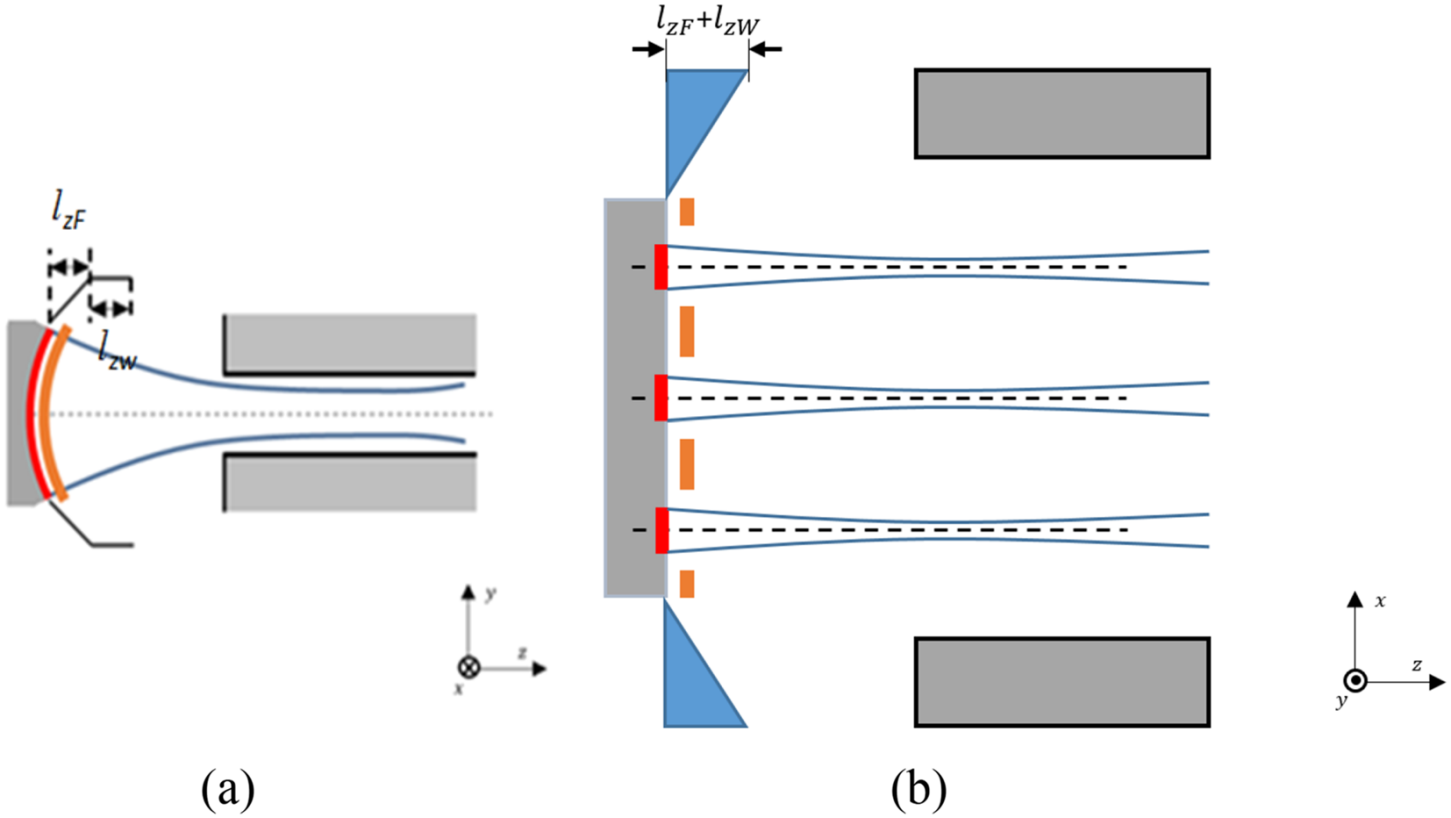
Schematic of the novel planar three-beam electron gun in both two transverse directions. (**a**) Front view. (**b**) Top view.

Figure 2 shows the schematic of the three-beam gun in both two transverse directions. It’s obvious that the outline of the three-beam gun is constructed identically to the sheet beam gun with the same profile except for the focusing electrode. In the following discussion, the centers of three beamlets are at x = -0.46 mm, y = 0 mm, at x = 0, y = 0, and at x = 0.46 mm, y = 0, respectively. Three-beam array emits electrons from the cathode that locates at z = 0, which will be transported along the positive direction of the z-axis.

**Figure 3:**
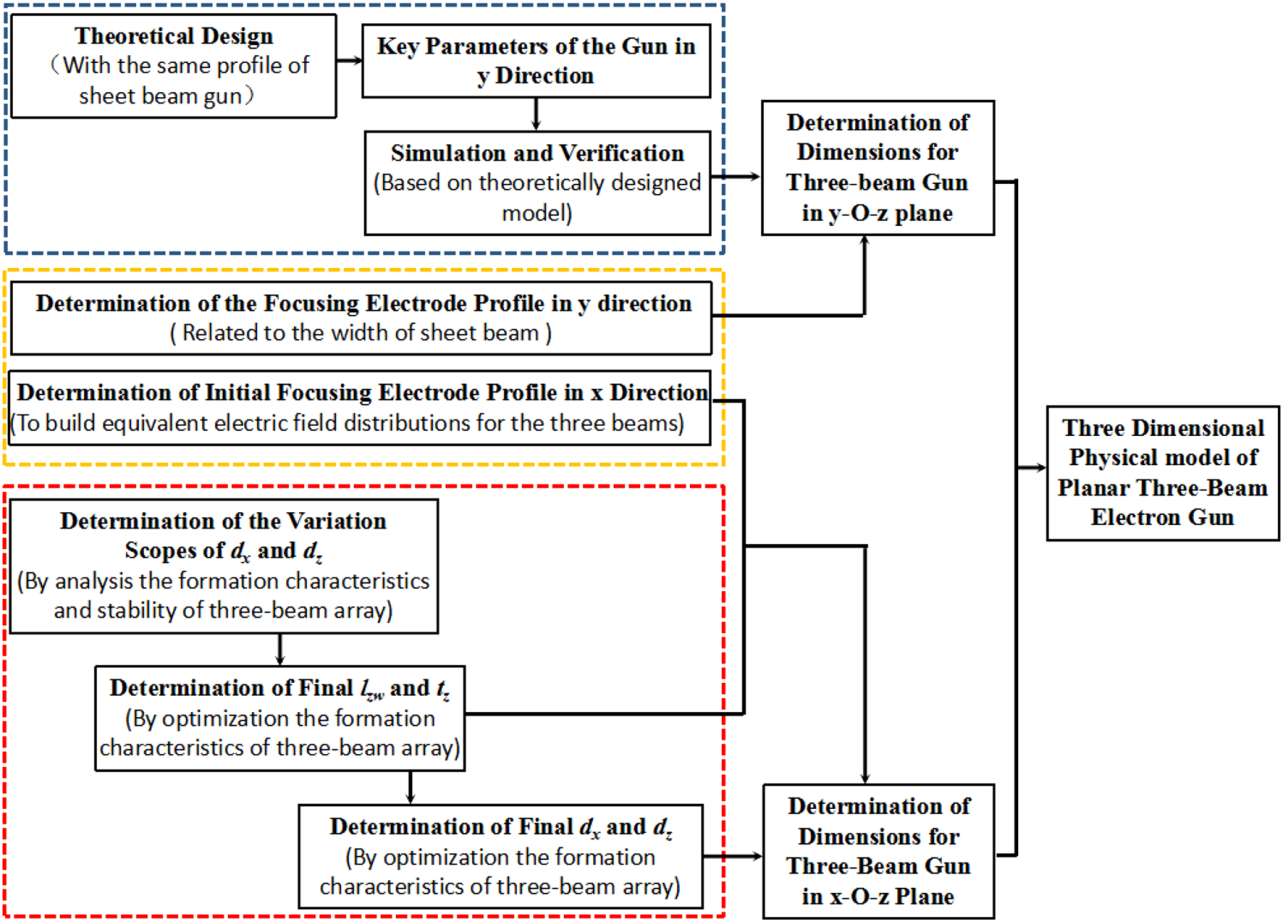
Logical diagram of the design guidelines for the novel planar three-beam electron gun.

Figure 3 shows the logical diagram that indicates the detailed design guidelines of the novel planar three-beam gun with narrow beam separation in this paper. Apparently, the design process of the planar three-beam gun can be simply divided into two steps. Firstly, the dimension of the three-beam gun in the y–O-z plane is determined based on the theory guideline for designing Pierce-type sheet beam gun as our work in reference^15^. In this step, several key parameters of the three-beam gun can be determined, including the half-angle $$\theta$$, curvature radius of cathode $$ R_{c}$$ and anode $$R_{a}$$, spacing from cathode to anode $${\text{d}}K_{a}$$, half-height of anode aperture $$r_{a}$$. Then the simulations are used to verify the key parameters and beam transport in y–O-z plane thoroughly. Secondly, the dimension of the three-beam gun in the x-O-z plane is determined based on the scopes analysis for the formation characteristics and stability of three-beam array with several parameters optimization, which includes the separation between adjacent grids wires $$d_{x}$$, the extension length of focusing electrode $$l_{zW}$$, the thickness of the control grids $$t_{z}$$,and the separation from cathode to grid $$d_{z}$$ focusing electrode. The results show that the determination of the profile of focusing electrode in two directions can be included in the two steps, respectively.

**Figure 4:**
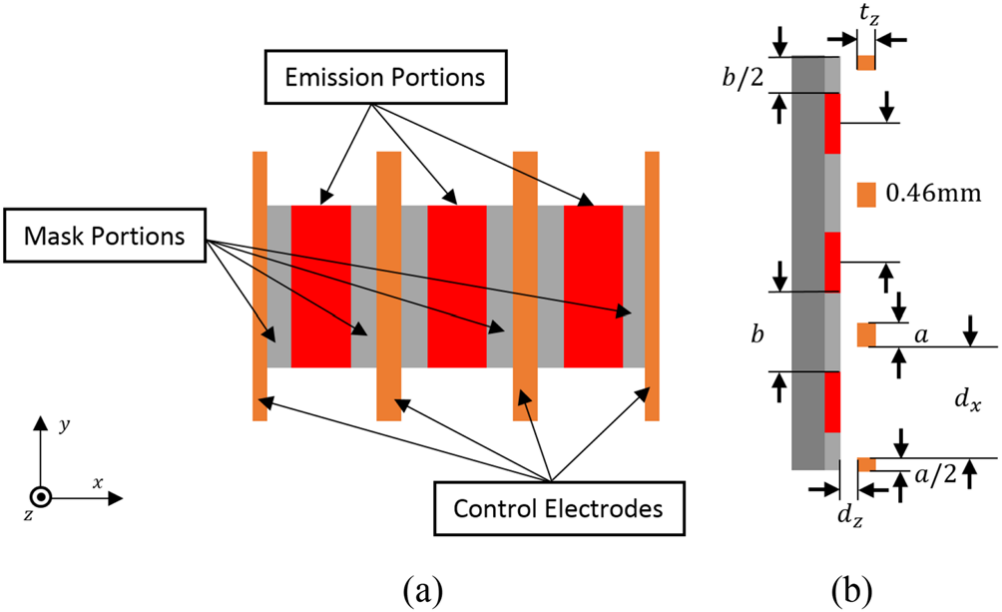
Configuration of the three-beam cathode combined with the control grids. (**a**) Right view. (**b**) Top view.

Figure 4 shows the configuration of the three-beam cathode combined with the control grids. Width (dimensions in x direction) of the side portions of the control electrodes (*a*/2) and the masks (*b*/2) equal half of their counterparts, respectively, which is located in the middle of the structure to construct the equivalent electric field distributions for the side beams and the central beam. To obtain the excellent beam transmission of the three-beam array, we’d like to keep a smaller beam-to-tunnel radial fill factor. The intended average radius at the beam waist is about 0.08 mm. Thus, half-height of the emission portion is about 0.32 mm with the selected beam compression factor of 4 in y direction. Half-width of each emission portion is chosen as 0.1 mm with compromising the lower emission current density and the focusing characteristics of the control grids. Finally, the cross-sections of each emission portion and the complete cathode are about $0.2{\text{ mm}} \times 0.64{\text{ mm}}$, and $1.38{\text{ mm}} \times 0.64{\text{ mm}}$, respectively. The compression factor of each beamlet in y direction is set to 4, which means that the current emission density of 117 A/cm^2^ is required for the cathode. Such high emission density can be satisfied with a new type of impregnated dispenser cathode using the active substance with a molar ratio of 26BaO·29SrO·8Sc_2_O_3_·7CaO·Al_2_O_3_, which can achieve the electron beam emission density of over 160 A/cm^2^ experimentally in reference^18^.

In part III of this paper, we show that the dimension of the three-beam gun in the y–O-z plane is determined based on the theory guideline for designing Pierce-type sheet beam gun as our work in reference^17^. Each electron beam formation comes from a narrow rectangle cathode, which is only compressed in y direction with a ratio of 4. However, in part IV, we constructed the desired electric field distribution by the focusing electrode combined with the control grids, which is different from conventional Pierce-type guns, attaining the same proper beam size in the x direction. As a result, the rectangle electron beam in the beam waist is formed with the same dimension in x and y direction. Thus, in our manuscript, we use the description of radium symbols, e.g., the *R* and *r* which are widely used in the design of the traditional round electron beam. Such a description only denotes that our designed three electron beams have the same dimension in x and y direction.

## Dimensions of the three-beam gun in y–O-z plane

In this section, several key dimensions of the three-beam gun in the y–O-z plane are determined based on the extended theoretical method used for designing sheet beam gun with the same profile in reference^17^. Simulation results show that the required length $l_{zW}$ of the focusing electrode and the beam throw distance $z_{m}$ are different according to the beam width in x direction. Thus, an appropriate range of $l_{zW}$ and $z_{m}$ will be estimated appropriately for the design of the three-beam gun.

**Figure 5:**
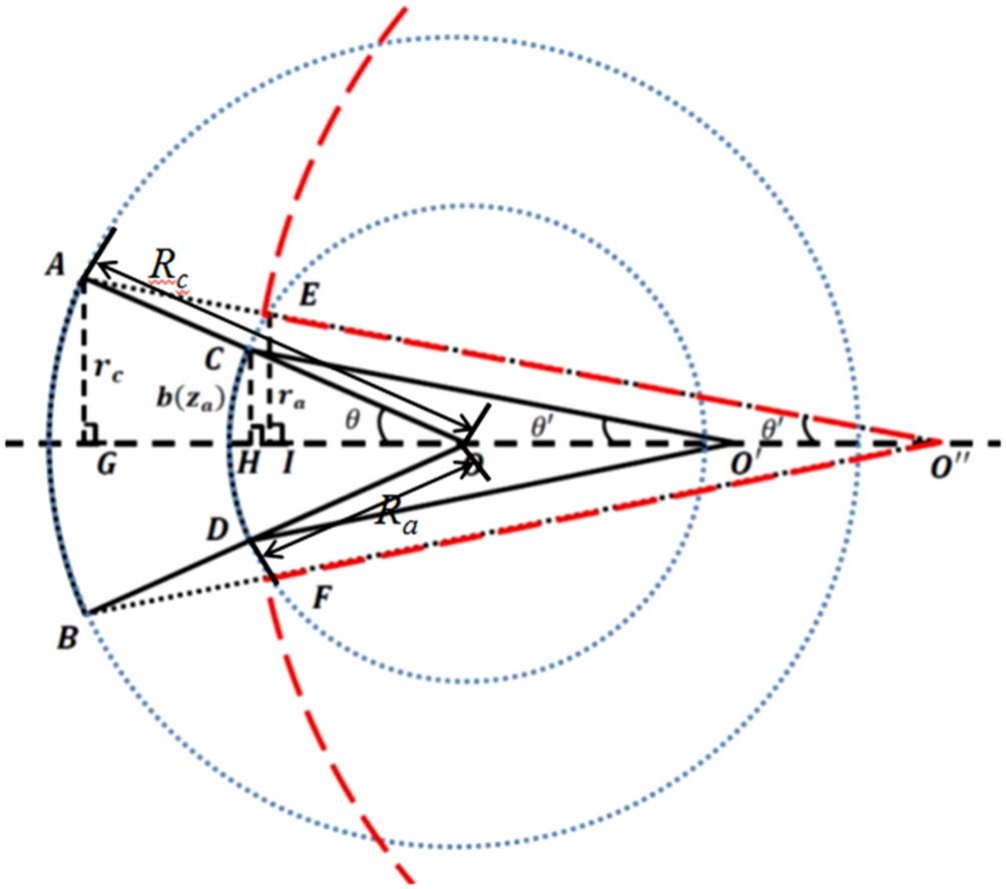
Schematic of the sheet beam electron gun with their parameters.

Figure 5 shows some important parameters for the design of sheet beam electron gun^17^ in this paper. Here we emphasize that $R_{{\text{c}}}$ and $R_{a}$ are the curvature radius of the cathode and the anode. $\theta$ is the half-angle, which means the angle between the cathode edge and the convergence point when the sheet beam passing through the anode aperture.$\theta ^{\prime}$ is the new half-angle under the influence of the divergence of the anode aperture. $\theta ^{\prime\prime}$ is the equivalent half-angle considering both the convergence of the initial sheet beam and the divergence of the anode aperture. $r_{c}$ is the half height of the cathode while $r_{a}$ is the half height of the anode aperture. It’s obvious that $\theta$ only depends on $R_{a}$ (or $R_{{\text{c}}}$) when $R_{{\text{c}}} /R_{a}$ is given.

### Determination of half-angle θ

**Figure 6:**
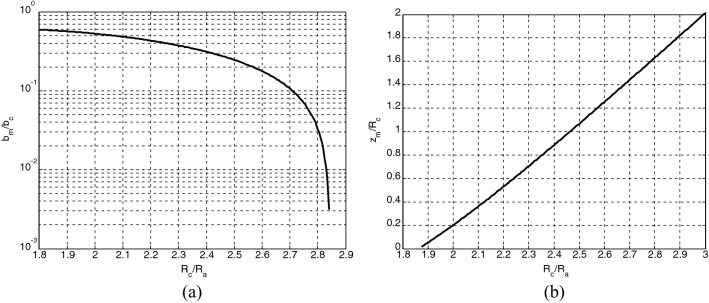
Design curves of sheet beam gun for (**a**) compression factor and (**b**) throw distance.

According to the designed three-beam gun model, the current of beam portion with unit width $I_{u}$ can be calculated as 0.75 A/mm according to reference^17^. The design curves of beam compression factor and throw distance for the sheet beam gun are shown in Fig. 6, respectively. According to Fig. 6(a) with the beam compression factor of 4, the value of the curvature radius ratio of the cathode to anode $R_{{\text{c}}} /R_{a}$ can be calculated as 2.464. Next, the L-B function $\left( { - \beta_{a} } \right)^{2}$ can be consulted from the chart of $\left( { - \beta } \right)^{2} \sim R_{c} /R$ [Table III in^19^] as 1.705. Then, substituting the value of $I_{u}$, $U_{a}$, $\left( { - \beta_{a} } \right)^{2}$ into

1 $$ I_{u} = 14.67 \times 10^{ - 6} \frac{\theta }{\pi }\frac{{U_{a}^{3/2} }}{{R_{a} \left( { - \beta_{a} } \right)^{2} }} $$

the curvature radius of anode $R_{a}$ can be expressed in $\theta$

2 $$ R_{a} = 11.924\theta $$

The curvature radius of cathode $R_{c}$ can be expressed as

3 $$ R_{c} \approx \frac{{b_{c} }}{\theta } $$

Combining (2) and (3) with considering the value of $R_{{\text{c}}} /R_{a}$, the half angle $\theta$ can be calculated as 5.983 degrees.

### Determination of other parameters for sheet beam gun

According to the following formulas (4), (5), (6), the value of $R_{c}$, $R_{a}$, and the spacing from cathode to anode ${\text{d}}K_{a}$ can be calculated as 3.070 mm, 1.246 mm, 1.814 mm, respectively.

4 $$ R_{c} = \frac{{b_{c} }}{{{\text{sin}}\theta }} $$

5 $$ R_{a} = \frac{{R_{c} }}{{R_{c} /R_{a} }} $$

6 $$ dK_{a} = \left( {R_{c} - R_{a} } \right) \cdot {\text{cos}}\theta $$

The throw distance $z_{m}$ can be obtained as 3.011 mm from Fig. 6(b) with the value of $R_{{\text{c}}} /R_{a}$.

According to the advanced method for anode aperture reconstruction in sheet beam gun, half-height of the reconstructed anode aperture $r_{a}$ can be calculated as 0.245 mm. Considering the correction of cylindrical aberration, the key parameters for sheet beam gun can be determined successfully.

### Simulation and verification

To verify the effectiveness of the theoretical method above for design sheet beam guns and study the characteristics of a focusing electrode, two sheet beam gun models with different cathode cross-sections are constructed using CST PS model^20,21^, which are of single emission portion (Model I) and the complete cathode (Model II) of the three-beam gun, respectively.

A focusing electrode in sheet beam gun is constructed with the electric field distribution for wedge-shaped converging radial flow. In our sheet beam gun model, the focusing electrode profile in the y direction is preliminary composed of two tiled metal walls which makes an angle of 67.5^0^ with the normal to the cathode followed by two parallel metal planes. Combined with 3D simulation software CST PS, the proper axial dimensions of the tiled parts in the two models are both $l_{zF} = 0.2 {\text{mm}}$, but of the parallel portions are $l_{zW} = 0.13 {\text{mm}}$ for Model I and $l_{zW} = 0.24 {\text{mm}}$ for Model II, respectively, when both of them are performed well.

**Figure 7:**
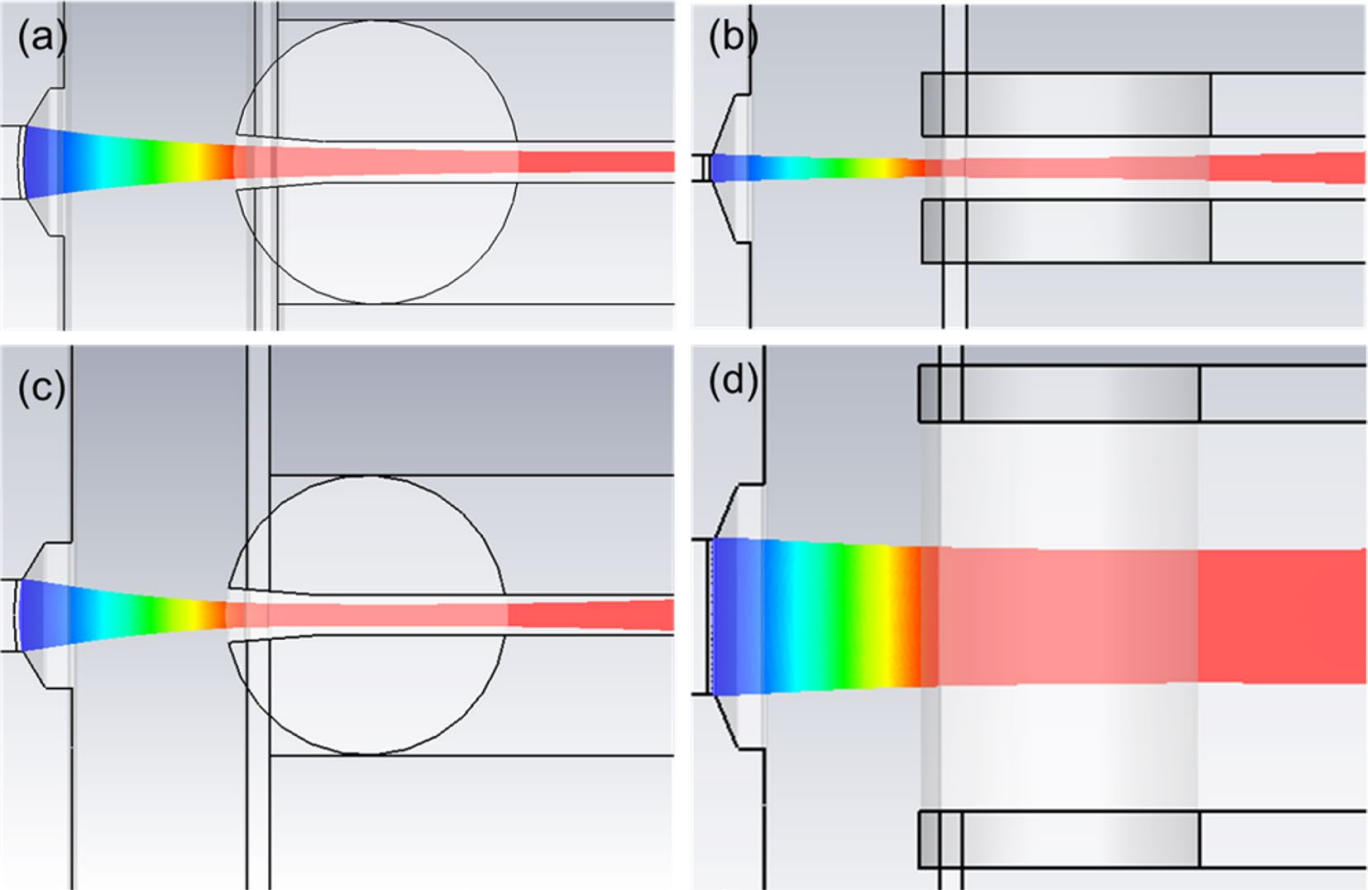
Beam trajectory in two transverse directions for the two models. (**a**) In the y–O-z plane of Model I. (**b**) In the x-O-z plane of Model I. (**c**) In the y–O-z plane of Model II. (**d**) In the x-O-z plane of Model II.

**Figure 8:**
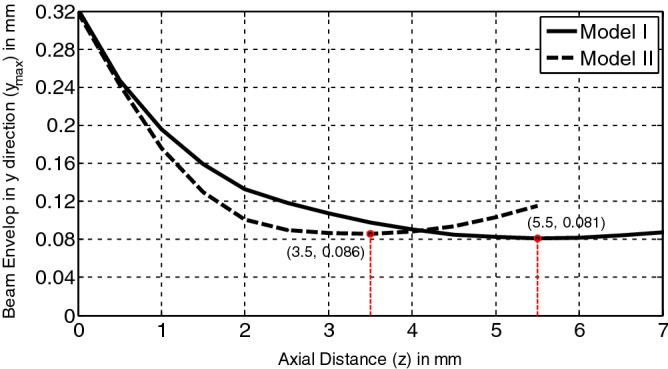
Compressions of beam envelops in *y* direction of the two models.

Simulated beam trajectories of the two models are shown in Fig. 7. Figure 8 compares the simulated beam envelops in the *y* direction of the two models. Apparently, both of the two sheet beams achieve their intended waist of 0.08 mm. However, the throw distances $z_{m}$ in the two models are quite different. A further throw distance, 5.5 mm, is more likely to take place in Model I (with a smaller beam width). However, the throw distance in Model II is about 3.5 mm.

The main reason for the differences above in $l_{zW}$ and $z_{m}$ in two models may be that the y components of spacing charge force in sheet beams are different with various beam width in x direction, which results from the influence of beam boundary distortion. A smaller y component for the space charge force is with the smaller width sheet beam due to a more serious influence of boundary distortion in it. Thus, the convergent effect of the focusing electrode needed in Model I should be less than it needed in Model II. Consequently, an intermediate convergent effect of the focusing electrode is needed in our three-beam gun, 0.13 mm $< l_{zW} <$ 0.24 mm. And the throw distance of each beamlet in our three-beam array may be more than 3.5 mm and less than 5.5 mm.

From the calculation, the beam current is about 0.155 A for Model I and 1.076 A for Model II corresponds to the unit beam current of 0.78 A/mm, which is close to the expected value 0.75 A/mm. Thus, the effectiveness of the above method for designing a sheet beam gun is verified and the key parameters will be used in the following three-beam gun construction.

## Determination of the dimensions for three-beam gun in x-O-z plane with beam formation stability

Based on the analysis, the overall outline of the novel three-beam gun in the x-O-z plane can be initially built according to the sheet beam gun in Model II. However, the focusing electrode profile should be unique for the three-beam gun with the reason of different electric field distributions required for beam compression of the three-beam array and the sheet beam. In the novel three-beam gun, the desired electric field distribution for three convergent beamlets is constructed by the focusing electrode combined with the control grids. Their characteristics decide the performance of the three-beam gun directly and effectively. In our model, the potentials of the focusing electrode and the control grids are set at the cathode potential to make the simplicity and stable operation of the gun. In this section, the profiles of focusing electrode in two transverse directions are determined, respectively, for the equivalence of three beamlets with good beam characteristics. Transmission verification of the beamlet with transverse velocity in the axial Brillouin magnetic field is carried out theoretically. At last, several key dimensions of the control grids are discussed based on the characteristic’s analysis of the three-beam array formation.

### Focusing electrode profile in x direction

Non-equivalence of the beamlets with different lateral positions in the three-beam array may cause quite a few problems in the following transport process under the magnetic field by the focusing structure. To generate three beamlets with similar properties, a novel focusing electrode is constructed in the paper, which can adjust the electrical field distribution in two transverse directions independently.

**Figure 9:**
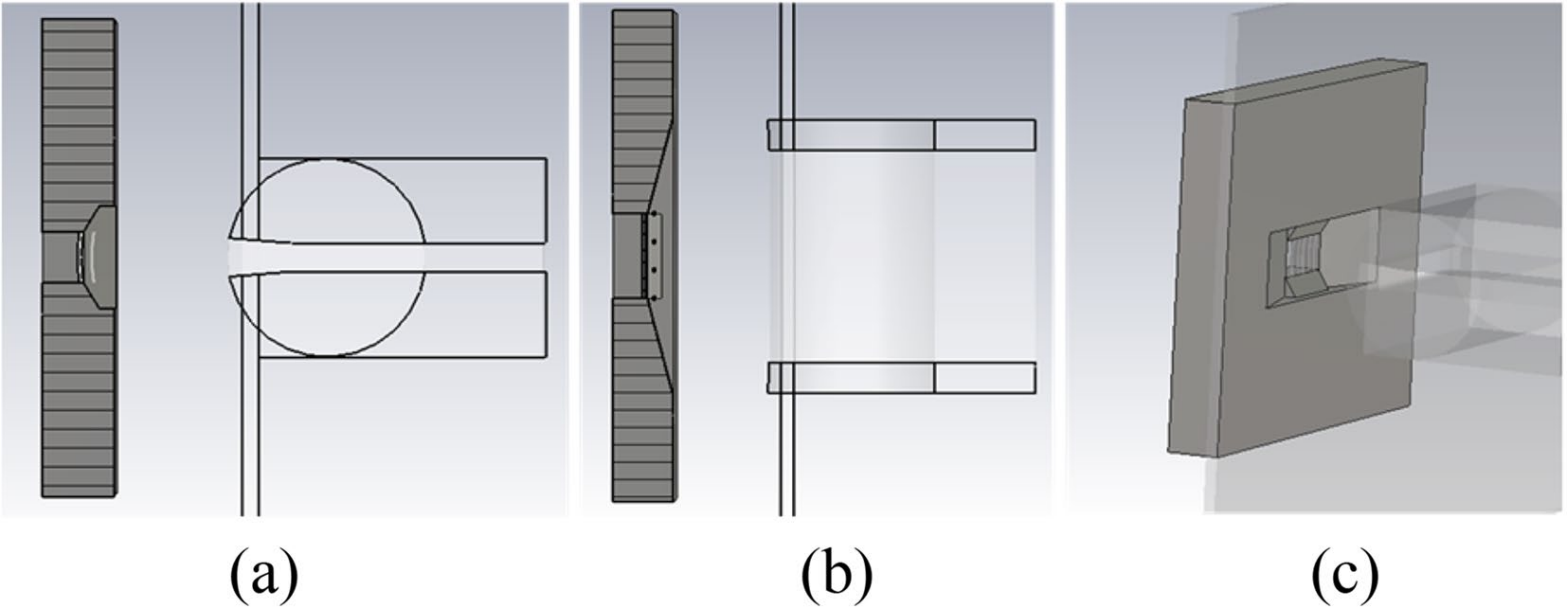
Configuration of the focusing electrode in the three-beam electron gun. (**a**) In the y–O-z plane. (**b**) In the x-O-z plane. (**c**) 3D view.

Figure 9 shows the configuration of the novel focusing electrode structure for the three-beam gun. The focusing structure profile in y direction is identical to the configuration in the sheet beam gun above. However, the profile in the x direction is without the two parallel metal plane parts, and the inclination angle of the focusing electrode in x direction called $\theta_{FX}$ is larger. By the much more numerical simulation analysis on various three-beam gun models, the beam current of each beamlet reaches an approximately identical value when $\theta_{FX}$ increase to a constant value. It’s about 1.13 times of 67.5^0^, e.g. the center point is 76.275^0^, which can make the three beamlets transport along their symmetric axes strictly. However, the side beam currents are less than the value of central one and the side beamlets are all tilting towards the gun center with beam transport, when $\theta^{\prime}$ is less than the critical point. This may be caused by a more convergent electric field distribution constructed by the focusing electrode in the x direction, which also constrains the electrons emission from the side emission surfaces.

### Determination of the variation scopes of ${\mathbf{d}}_{{\mathbf{x}}}$ and ${\mathbf{d}}_{{\mathbf{z}}}$

The preliminary determinations of the variation scopes of transverse dimensions $d_{x}$ and axial position $d_{z}$ of the control grids will facilitate the determinations of the other parameters and the design work of the novel three-beam gun, in Fig. 2. Thus, the variations of the two parameters are primarily discussed based on their effects on the throw distances in both two transverse directions.

**Figure 10:**
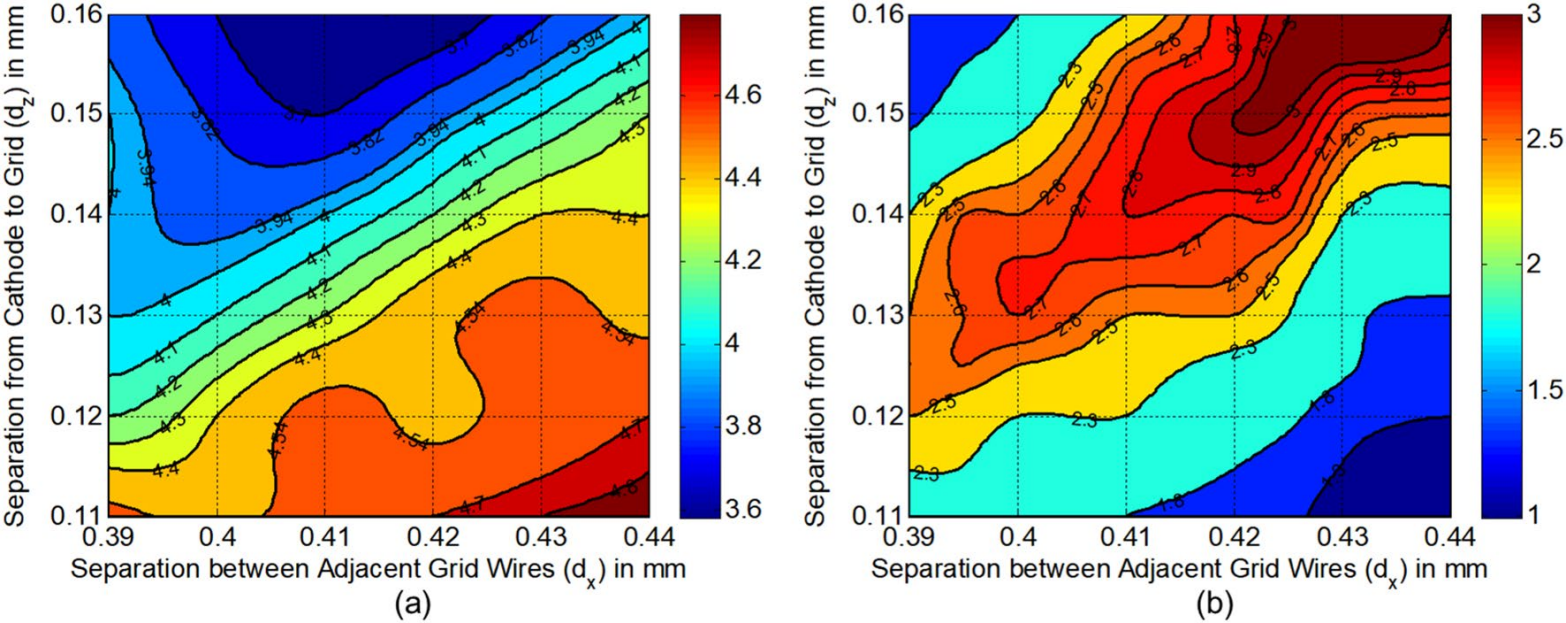
(**a**) Variation of throw distance in y direction $Z_{mY}$ with both $d_{x}$ and $d_{z}$. (**b**) Variation of throw distance in x direction $Z_{mX}$ with both $d_{x}$ and $d_{z}$.

Figure 10(a) gives the variation of throw distance in the y direction $Z_{mY}$ with both $d_{x}$ and $d_{z}$. In our calculation, we have selected the calculation points for $d_{x}$ from 0.39 to 0.44 mm, and $d_{z}$ from 0.11 to 0.16 mm with the small intervals of 0.002 mm, so that there are much of enough original data which can express the characteristics and tendency in all the similar figures in our paper. Also, we have used cubic spline interpolation in plotting all the figures with MATLAB to achieve good smoothness for all the curves in these figures. From the results, it’s clear that $Z_{mY}$ almost remains constant while keeping the variation ratio of $d_{x}$ and $d_{z}$ unchanged. And the linear change range of $Z_{mY}$ is mainly from 4.0 to 4.4 mm. Besides, the results verify the deduction in the following subsection that throw distance of each beamlet for three-beam may be more than 3.5 mm and less than 5.5 mm.

Figure 10(b) gives the variation of throw distance in the x direction $Z_{mX}$ with both $d_{x}$ and $d_{z}$. The models with $Z_{mX} < 2.3$ in the top left corner are invalid due to the trajectories cross each other under the stronger convergent function in the x direction. With considering the calculated spacing from cathode to anode $dK_{a}$ = 1.814 mm, the models with $Z_{mX} < 1.8$ in the bottom right corner are invalid due to the beam waist in the x direction hasn’t emerged into anode aperture. Thus, the upper limit of $d_{x}$ and $d_{z}$ are selected at 0.44 mm and 0.16 mm, respectively, to keep a wider valid range of data. Besides, $Z_{mX}$ is shrunk dramatically with minimizing $d_{z}$ and $d_{x}$ simultaneously at a constant ratio of $d_{x}$ and $d_{z}$, but $Z_{mY}$ almost keeps constant according to Fig. 10(a). To facilitate the design work and matching process of the magnetic focusing system, the three-beam gun with smaller waists separations among the two transverse directions are desired. Thus, the lower limits of $d_{x}$ and $d_{z}$ should not be too small. Consequently, the proper region of $d_{x}$ and $d_{z}$ are determined as to 0.39 mm $\sim$ 0.44 mm and 0.11 mm $\sim$ 0.16 mm, respectively.

### Determination of extension length of focusing electrode ${\varvec{l}}_{{{\varvec{zW}}}}$ and ${\varvec{t}}_{{\varvec{z}}}$

Throughout the analysis on focusing electrode above, all dimensions of the focusing electrode in the novel three-beam electron gun are determined except the parameter $l_{zW}$, which will be discussed in this subsection combined with the consideration of beam transport characteristics on both x and y directions. Control grids that serve as the ‘focusing electrode’ in the x direction control the formation and transport of the beamlets in x direction. The thickness of the control grids $t_{z}$ is also selected in this subsection based on the beam transport characteristics analysis for both transverse directions.

**Figure 11:**
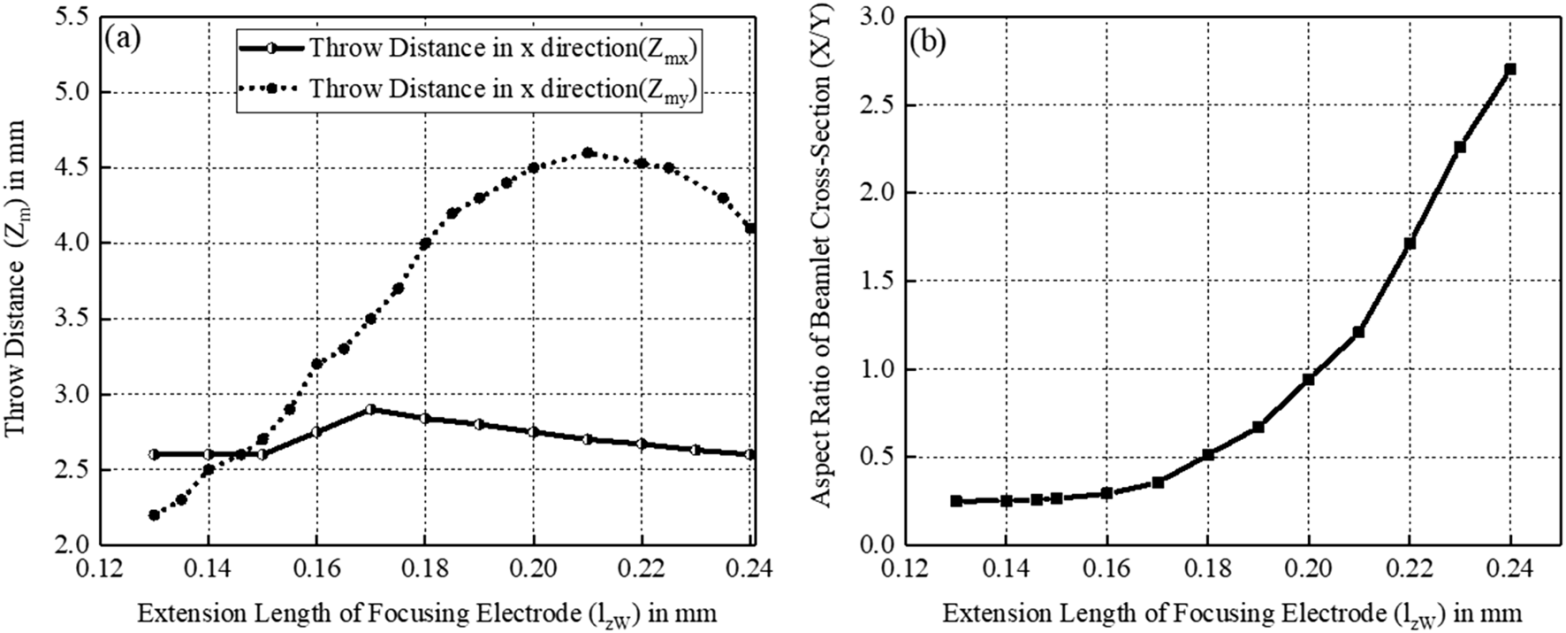
(**a**) Variation of throw distances in both *x* and *y* directions with $l_{zW}$. (**b**) Variation of aspect ratio of beam cross section at beam waist in *y* direction with $l_{zW}$.

From the above analysis, the three-beam gun model with intermediate values of $d_{x}$ = 0.415 mm and $d_{z}$ = 0.13 mm is selected to study the axial characteristics of focusing electrode and control grids, including $l_{zW}$ and $t_{z}$. Figure 11(a) shows the variation of throw distances in both *x* and *y* directions with $l_{zW}$. Figure 11(b) shows the variation of the aspect ratio (X/Y) of beam cross section at beam waist in *y* direction with $l_{zW}$. According to Fig. 11(a), the throw distances in both transverse directions meet in the gun model with $l_{zW }$ = 0.146 mm. However, according to Fig. 11(b), the aspect ratio of beam cross-section in this model is about 0.257 that close to the aspect ratio of cathode 0.313 and is far from the desired circular beam. According to Fig. 11(a), it’s obvious that the throw distance in y direction $Z_{my}$ is raising with the extended length $l_{zW}$ of focusing electrode since the incidence slope of boundary electrons are increased. The reason for the drop of $Z_{my}$ at $l_{zW}$ = 0.24 mm is that electron trajectories from different layers cross each other. However, the throw distance in y direction $Z_{mx}$ is relatively insensitive to $l_{zW}$. To facilitate the design work of the following magnetic focusing system, the beam array with a closer beam waist in both transverse direction is desired, which means a shorter $l_{zW}$ according to Fig. 11(a). Besides, the value of $l_{zW}$ should not be too small from Fig. 11(b) to make the cross-section at the beam waist close to be circular. Thus, the value of $l_{zW}$ is compromised as 0.19 mm with the aspect ratio of beam cross-section at the waist about 0.7.

**Figure 12:**
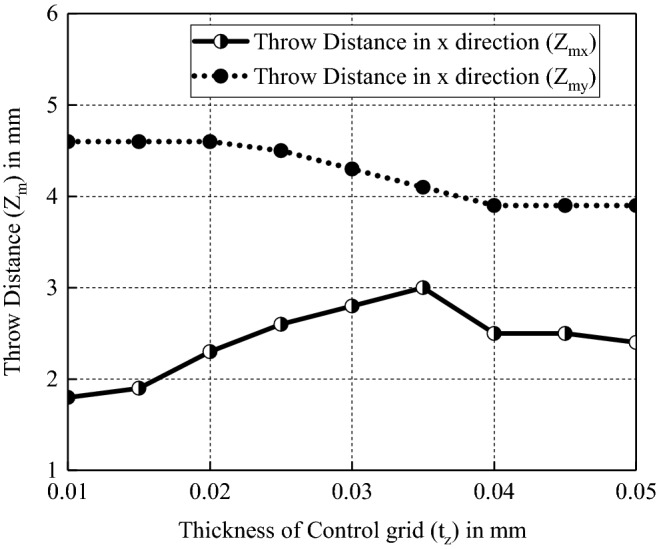
Variation of throw distances in both *x* and *y* directions with $t_{z}$.

After determining the value of $l_{zW}$, we explored the influence of the value of $t_{z}$ on the throw distances in both *x* and *y* directions, as shown in Fig. 12. It’s obvious that the throw distance in the x direction $Z_{mx}$ is increasing with $t_{z}$ firstly and begins to drop beyond the critical point of $t_{z}$ = 0.035 mm. But the throw distance in the y direction $Z_{my}$ always decreases as $t_{z}$ increases. Thus, a greater thickness of control grids but no more than 0.035 mm would be like for a narrower axial separation between two beam waists. Considering the stability of the gun and the machining error, $t_{z}$ = 0.03 mm is selected as the thickness of the control grid.

### Determination of ${\mathbf{d}}_{{\mathbf{z}}}$ and ${\mathbf{d}}_{{\mathbf{x}}}$ with beam formation stability

**Figure 13:**
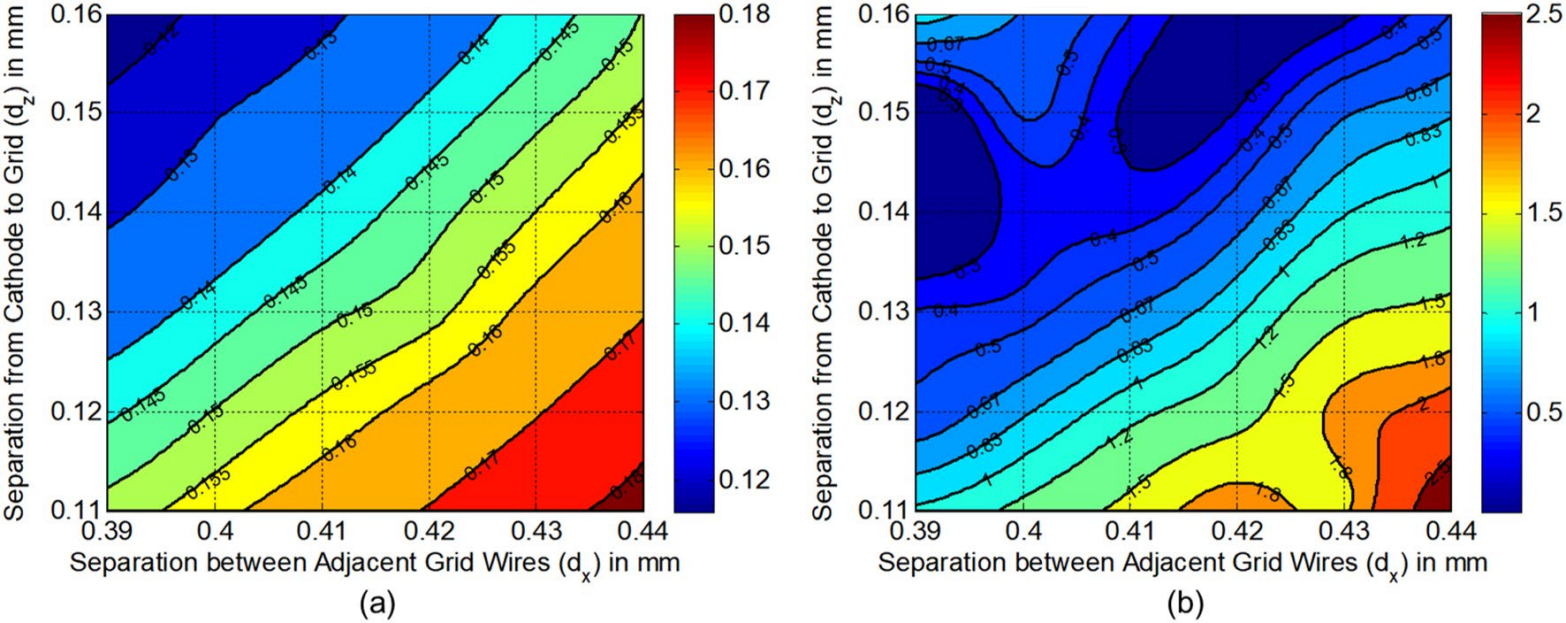
(**a**) Variation of beam current with $d_{z}$ and $d_{x}$. (**b**) Variation of beam aspect ratio (x/y) at the beam waist in y direction with $d_{z}$ and $d_{x}$.

Based on the structure of the three-beam gun determined, the value of $d_{x}$ and $d_{z}$ are in the scope of 0.39 mm $\sim$ 0.44 mm and 0.11 mm $\sim$ 0.16 mm, respectively. In this range, Fig. 13(a) shows the variation of beam current and stability with $d_{z}$ and $d_{x}$. It’s found that the beam current is decreasing with enhancing $d_{z}$, or minimizing $d_{x}$, and the beam current almost unchanged and kept more stability with broadening $d_{z}$ and $d_{x}$ simultaneously. According to the design specification, the values of $d_{z}$ and $d_{x}$ that keeps the beam current varies in the range of 0.145 A $\sim$ 0.155 A could be selected easily.

Figure 13(b) shows the variation of beam aspect ratio (x/y) at the beam waist in the y direction with $d_{z}$ and $d_{x}$. It can be seen that the aspect ratio mainly increases with minimizing $d_{z}$, or enhancing $d_{x}$ in the scope where the beam current is acceptable. Considering the stable beam transport in the following magnetic field, the beam aspect ratio is better limited from 0.67 to 1.

When the proper combinations of $d_{z}$ and $d_{x}$ are selected, Fig. 11(a) and (b) should be taken into consideration to verify if the model is with a small waist separation in the two transverse directions.

### Construction and simulation

**Figure 14:**
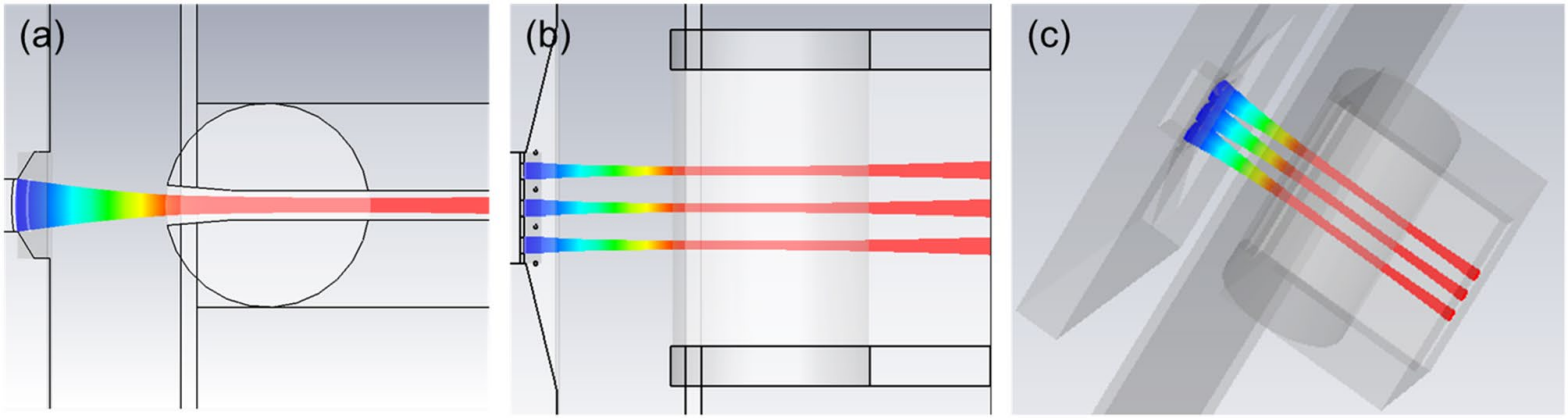
Beam trajectory of the novel planar three-beam gun. (**a**) In the y–O-z plane. (**b**) In the x-O-z plane. (**c**) 3D view.

**Figure 15:**
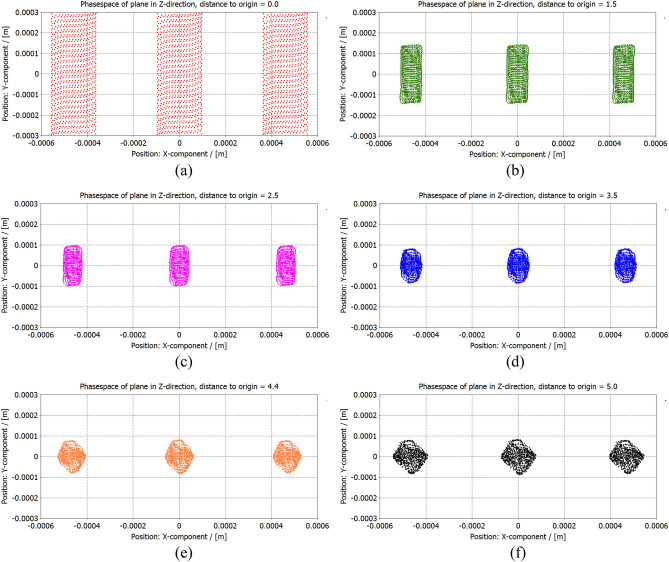
Beam cross-section along the direction of beam transport. (**a**) z = 0, (**b**) z = 1.5 mm, (**c**) z = 2.5 mm, (**d**) z = 3.5 mm, (**e**) z = 4.4 mm, (**f**) z = 5.0 mm.

To guarantee the good beam characteristics and satisfy the demands from the high frequency circuit, the three-beam array with the beam waist aspect ratio from 0.67 to 1 and the beam current from 0.145 A to 0.155 A would like to be taken into consideration. The combination of $d_{z}$ = 0.13 mm and $d_{x}$ = 0.415 mm is finally adopted in our design for the novel planar three-beam electron gun with CST simulation^20,21^. Figure 14 shows the beam trajectory from different perspectives. Apparently, the three-beam array is well generated and compressed on both two transverse directions. Figure 15 shows the beam cross-section along the direction of beam transport. The three-beam array achieves the waist in the x direction at about 2.5 mm away from the cathode, and achieves the waist in y direction at about 4.4 mm away from the cathode. The profiles at beam waists in the x direction and the y direction are indicated in (c) and (e), respectively. The beam aspect ratio at the waist in y direction is about 0.83. And the simulated beam current is about 3 $\times$ 0.153 A.

For the design of our three-beam electron gun with small distances between cathode, focusing electrode and anode, the high voltage will cause the breakdown due to the strong electric field strength. As we know, with the high vacuum (~ 10^–7^ Pa–10^–8^ Pa), the breakdown field strength is more than 10^6^ V/cm as the experienced data. However, from the electric field distribution in our designed three-beam electron gun with CST PS, we know the maximum electric field of the whole structure is about 3 × 10^5^ V/cm (the maximum electric field appears in the four corners of the edge the focusing electrode), which is smaller than the breakdown electric field of 10^6^ V/cm in high vacuum. Thus, the breakdown in our electron gun cannot happen.

## Conclusion

Only by developing in the direction of planarization, miniaturization and integration, can the vacuum electronic devices be expected to break through the limitations of existing mechanisms, and thus achieve leapfrog development in the THz band. In this paper, methodologies and guidelines for designing the novel planar distributed three-beam electron gun are presented with narrow beam separations, which can be used in such type of narrow separation beam electron gun as one of the general method. The physical model of our novel planar three-beam gun with optimal characteristics is verified using simulation in the 3D software, the beam current of 3 $\times$ 0.15 A, an average beam radius of 0.08 mm for each beamlet waist is obtained successfully with a beam compression ratio of 4. Consequently, the designed three-beam electron gun will be used in our fundamental mode staggered double vane TWT in W-band with high output power later. Moreover, such planar distributed multi-beam with independent beam tunnels and singly convergent beamlets, can achieve high output power by sustaining the high total beam current, and avoids the over-mode beam tunnels comparing with the sheet beam devices. Such novel scheme for the planar multiple beam combined with the planar interaction circuit are not only suitable for the planar distributed vacuum electron devices, e.g. traveling wave tube, extended interaction klystron and oscillator, in widely range for the millimeter wave and THz regime, but also it can meet the trend of planarity, miniaturization and integration for the development of next generation of vacuum electron devices with good potential for the high output power and broad bandwidth in the future.
